# An exploratory investigation of glucocorticoids, personality and survival rates in wild and rehabilitated hedgehogs (*Erinaceus europaeus*) in Denmark

**DOI:** 10.1186/s12862-021-01816-7

**Published:** 2021-05-22

**Authors:** Sophie Lund Rasmussen, Otto Kalliokoski, Torben Dabelsteen, Klas Abelson

**Affiliations:** 1 Wildlife Conservation Research Unit, Department of Zoology, The Recanati-Kaplan Centre, University of Oxford, Tubney House, Abingdon Road, Tubney, Abingdon, OX13 5QL UK; 2 Department of Chemistry and Bioscience, Aalborg University, Fredrik Bajers Vej, 7H, 9220 Aaborg, Denmark; 3 Department of Biology, Section for Ecology and Evolution, University of Copenhagen, Universitetsparken 15, Building 12, 2100 Copenhagen Ø, Denmark; 4 Department of Experimental Medicine, University of Copenhagen, Blegdamsvej 3, 2200 Copenhagen N, Denmark

**Keywords:** Cortisol, Corticosterone, Stress, Wildlife rehabilitation, Wildlife conservation, Behaviour

## Abstract

**Background:**

The European population of hedgehogs (*Erinaceus europaeus*) is declining. It is therefore essential to optimise conservation initiatives such as the rehabilitation of sick, injured and orphaned hedgehogs. Wild animals placed in captivity may be prone to chronic stress, potentially causing negative health effects. Therefore, the effects of these rehabilitation efforts should consequently be evaluated. Furthermore, hand-raising orphaned hedgehogs is a laborious and costly task, and it is therefore relevant to document whether they have equal post release survival rates compared to their wild conspecifics.

The objectives of this research were therefore to conduct an exploratory study of glucocorticoid levels in hedgehogs from different backgrounds and compare the post release survival of translocated, rehabilitated and wild, juvenile hedgehogs as well as the possible effect on survival of differences in shy or bold behaviour (personality) exhibited by individuals.

**Results:**

We measured glucocorticoid levels in 43 wild-caught (n = 18) and rehabilitated (n = 25) hedgehogs and compared the post release survival and spatial behaviour of 18 translocated juvenile hedgehogs (eight hand-raised and ten wild) until hibernation. The possible effect on survival of differences in shy or bold behaviour (personality) exhibited by 17 juvenile individuals (seven hand-raised and ten wild) was also examined.

Rehabilitated individuals and females had higher levels of faecal corticosterone metabolites compared to wild individuals and males, respectively. Rehabilitated individuals showed higher levels of saliva corticosterone than wild. The personality tests labelled 13 individuals as shy and 11 as bold. Post release survival was 57% for rehabilitated and 50% for wild individuals. Neither background nor personality affected post release survival. Home range measures were 3.54 and 4.85 ha. Mean dispersal length from the release sites was 217 ± 100 m.

**Conclusion:**

The higher levels of corticosterone observed in rehabilitated compared to wild hedgehogs calls for consideration of the duration of admission to wildlife rehabilitation centres to reduce stress levels in the patients.

Hand-raised juveniles appear to have the same prospects as wild, and personality does not seem to affect post release survival in hedgehogs, indicating that hand-raising of orphaned juvenile hedgehogs is a relevant contribution to the conservation of this species.

**Supplementary Information:**

The online version contains supplementary material available at 10.1186/s12862-021-01816-7.

## Background

### Conservation and status of the European hedgehog

The western European hedgehog (*Erinaceus europaeus*) is found on the British Isles and Continental Europe, from Iberia and Italy in the south to Scandinavia in the north, as well as on New Zealand. It is widely distributed and can survive across a wide range of habitat types [1, 2]. However, investigations on both national and local scales have documented declines, or expressed concerns about decline, of the hedgehog populations in several western European countries [3–10]. The suspected reasons for the decline include habitat loss and fragmentation, intensified agricultural practices, inbreeding, road traffic accidents, lack of biodiversity and suitable nest sites in residential gardens, molluscicide and rodenticide poisoning, and badger predation [4, 11–23]. In Denmark, where this study occurred, hedgehogs become active after hibernation in mid-April to mid-May [22, 24, 25]. The juveniles are typically born from late July onwards and become independent around mid-September [26]. During mild autumns, second litters have been observed [22]. Hibernation is usually initiated from late September for adult males, late October for adult females and mid-November for young of the year [27]. However, hibernation may be postponed if the conditions are mild and food is available [22].

During the past 30 years, the rehabilitation of sick, orphaned, or injured wild hedgehogs has become an established practice in many western European countries. Denmark has several working hedgehog rehabilitation centres, where volunteers care for hedgehogs and release them back into the wild after recovery. The extent of hedgehog rehabilitation in Denmark is quite comprehensive, with the three largest organisations taking approximately 3200 hedgehogs into care during a year (pers. comm. Dyrenes Beskyttelse, Pindsvinevennerne i Danmark and Pindsvine Plejerne). Yet, Danish authorities have only recently established legal frameworks and monitoring programs for the practice of wildlife rehabilitation [28].

There are currently no monitoring programmes in Denmark tracking population numbers, however the data from other European countries is concerning. Conservation actions to preserve the species in the wild should thus be optimised and initiated across Europe.

### Wildlife rehabilitation and the effect of stress

The rehabilitation of orphaned, sick or injured wildlife followed by their release back into the wild is an important aspect of the conservation of threatened wildlife [29, 30]. However, when wild animals are placed in captivity, e.g. at a wildlife rehabilitation centre, they encounter a novel, confined and unpredictable environment, which often includes handling and close proximity to humans [31]. These conditions cause physiological stress responses in a range of species [32–36], which can have severe effects on their health [37–40]. Previous research has documented that chronic physiological stress can have detrimental consequences that may affect the recovery process [41], such as reduction in immuno-responsiveness [42] and body mass [43, 44]. Physiological stress may even cause death from e.g. capture myopathy, which can occur in several different forms with the more acute being capture shock syndrome (sudden death at capture or a few hours after capture) or acute/ataxic myoglobinuric syndrome (death a few hours to a few days after capture) [45]. It is therefore essential to understand the causes, risks and effects of physiological stress in the wildlife species one wishes to rehabilitate to improve animal welfare and survival during the care and captivity, and thereby eventually enhance the conservation success. This is especially important when handling a species such as the European hedgehog, which is undergoing a documented decline.

### Measuring stress in animals

Glucocorticoid (GC) levels, measured in a number of matrices (blood, saliva, urine, faeces, milk, etc.) can be used as a proxy measure of stress [46] since physiological and psychological stress are known to reliably increase circulating GC concentrations. Although, not a perfect measure of stress (there are numerous situations known to increase GC levels, but which are not considered stressful, e.g., sexual behaviour [47]), there is a considerable body of literature demonstrating the usefulness of assessing GC levels in captive and wild populations of wildlife. In the present investigation, corticosterone levels were measured in both saliva (as has previously been done for many species ranging from guineas pigs [48] to elephants and rhinoceros [49]) and (as corticosterone metabolites) in faeces (as has previously been done in a range of species from rats [50] to elephants [51]).

When analysing corticosterone or cortisol in faeces, it is not merely the steroids themselves that are quantified, but instead a plethora of immunoreactive metabolites produced in the liver during glucocorticoid metabolism, including cortisone/dehydrocorticosterone [52]. In saliva on the other hand, intact corticosterone and cortisol is measured. Thus, for the faecal samples, the term faecal corticosterone (or cortisol) metabolites (FCM) is used, and for saliva samples corticosterone and cortisol.

### Measuring personality in animals

Personality affects how individuals react to challenging situations [53] and may influence the post release survival of captive-reared mammals, as shown in a study by Bremner-Harrison et al. [54]. The personality of juvenile, captive-bred swift foxes was assessed and its influence on post release survival was scrutinised. The study revealed that bolder individuals were less suited for release if success was measured as post release survival, and it was suggested by the authors that the future selection of release-candidates based on personality should enhance the success on reintroduction programmes [54]. It is posited that the shyness/boldness of individuals can be estimated by analysing how they explore a novel environment or arena, and by measuring their latency to approach a novel object in a familiar environment [54–57]. Previous studies have demonstrated the existence of a shy–bold gradient in natural populations, and some have furthermore quantified the fitness consequences of personality [54, 58–60]. Personality can affect fitness through reproductive success and survival [54, 61, 62]. Population levels of boldness are subject to natural selection [63], which is why released individuals with inappropriate levels of boldness may suffer reduced fitness in the wild [54]. Therefore, when using rehabilitation of orphaned hedgehogs as a conservation effort for the species in general, it may be relevant to consider the personality of the individuals when deciding which release sites to use, since it may affect post release survival. This is particularly important with juveniles as their expected survival rate is low in general [60]. Previous studies have estimated survival probabilities for juvenile, Scandinavian hedgehogs ranging between 0.31 and 1.00 depending on the age and period of time in which they were studied [22, 25, 26, 64–66].

### Post release monitoring of rehabilitated hedgehogs

Previous research has investigated the post release survival and spatial behaviour of rehabilitated hedgehogs [30, 67–73], and some have included wild individuals for comparison [30, 70, 73] or described the survival of wild, translocated individuals [74]. However, few studies have directly compared the survival of rehabilitated and wild individuals, where both groups had been translocated [30].

Post release survival of rehabilitated hedgehogs have been found to range between 25 and 82% depending on the sample size (n = 4–34), age of the individuals (juveniles < 1 year or adults), time of year and duration of the studies (n = 3–22 weeks) [30, 67–74]. In two studies of rehabilitated, juvenile hedgehogs in the UK released during spring, the post release survival was 58% (n = 12, age = approximately 20 weeks, duration = 5–8 weeks from April) [69] and 77% (n = 13, age = approximately 20 weeks, duration = 6 weeks from April to June)[67]. The post release survival of rehabilitated, juvenile hedgehogs released in the UK during summer was 83% after 2 weeks, 75% after 4 weeks, 42% after 8 weeks and down to 25% 15 weeks post release (n = 12, age = autumn juveniles < 1 year released in June, duration = 15 weeks) [71].

In a study comparing five different groups of adult hedgehogs (local wild, local translocated wild, translocated rehabilitated, directly translocated wild from the Uist Islands (< 6 days in captivity) and translocated wild from the Uist Islands (> 1 month in captivity)), Molony et al. (2006) [30] discovered that the local wild hedgehogs had a significantly higher survival rate (94.7 ± 0.2%) than individuals in the rehabilitated translocated (73.1 ± 1.1%), directly translocated (40.9 ± 1.2%) and local translocated wild groups (63.6 ± 0.9%), and that the survival probability of translocated hedgehogs (having spent > 1 month in captivity) (81.8 ± 0.7%) was significantly greater than that for directly translocated individuals. Yarnell et al. (2019) [73] found no significant difference between the survival of wild and rehabilitated hedgehogs during the first 150 days after release of the rehabilitated individuals.

Morris and Warwick (1994) [69] recorded that three out of twelve rehabilitated, juvenile individuals dispersed up to 2 km away from the release site during the study period. The rest remained in the release area. Morris (1997) [67] described how all thirteen rehabilitated, juvenile hedgehogs remained within 400 m of the release point for at least a month post release, after which five hedgehogs dispersed, travelling at least 400 m and up to 5.2 km from the release point. Reeve (1998) [71] found that all surviving rehabilitated, juvenile individuals released in a rural woodland area dispersed from the release site during the 15 weeks of study, with a mean distance of 3 km, and the nearest animal found 1476 m from the release point. All individuals moved to areas of human habitation. In contrast, two individuals released into an urban area did not disperse far from the release site [71]. Molony et al. (2006) [30] found that there was a significant difference in the mean distance from the release site to the last known location after 8 weeks between the directly translocated wild group (directly translocated from the Uist Islands (< 6 days in captivity)), which travelled the largest mean distance (0.69 ± 0.82 km) compared to the rehabilitated translocated group (0.31 ± 0.33 km) and the translocated wild group (translocated from the Uist Islands (> 1 month in captivity)) (0.56 ± 0.45 km). The dispersal distance of the local wild group was 0.15 ± 0.14 km and 0.22 ± 0.18 km for the local, translocated wild group of hedgehogs.

### Aims

The objectives of the present study were:

1. To conduct an exploratory study of glucocorticoid levels in European hedgehogs from different locations, with different health status and backgrounds (wild and rehabilitated).
2. To quantify personality in European hedgehogs, measured as shyness-boldness, and to estimate the possible effects of personality on post release survival.
3. To measure and compare the post release survival of translocated, rehabilitated and wild, juvenile European hedgehogs.

## Results

### Salivary corticosterone levels

**Fig. 1:**
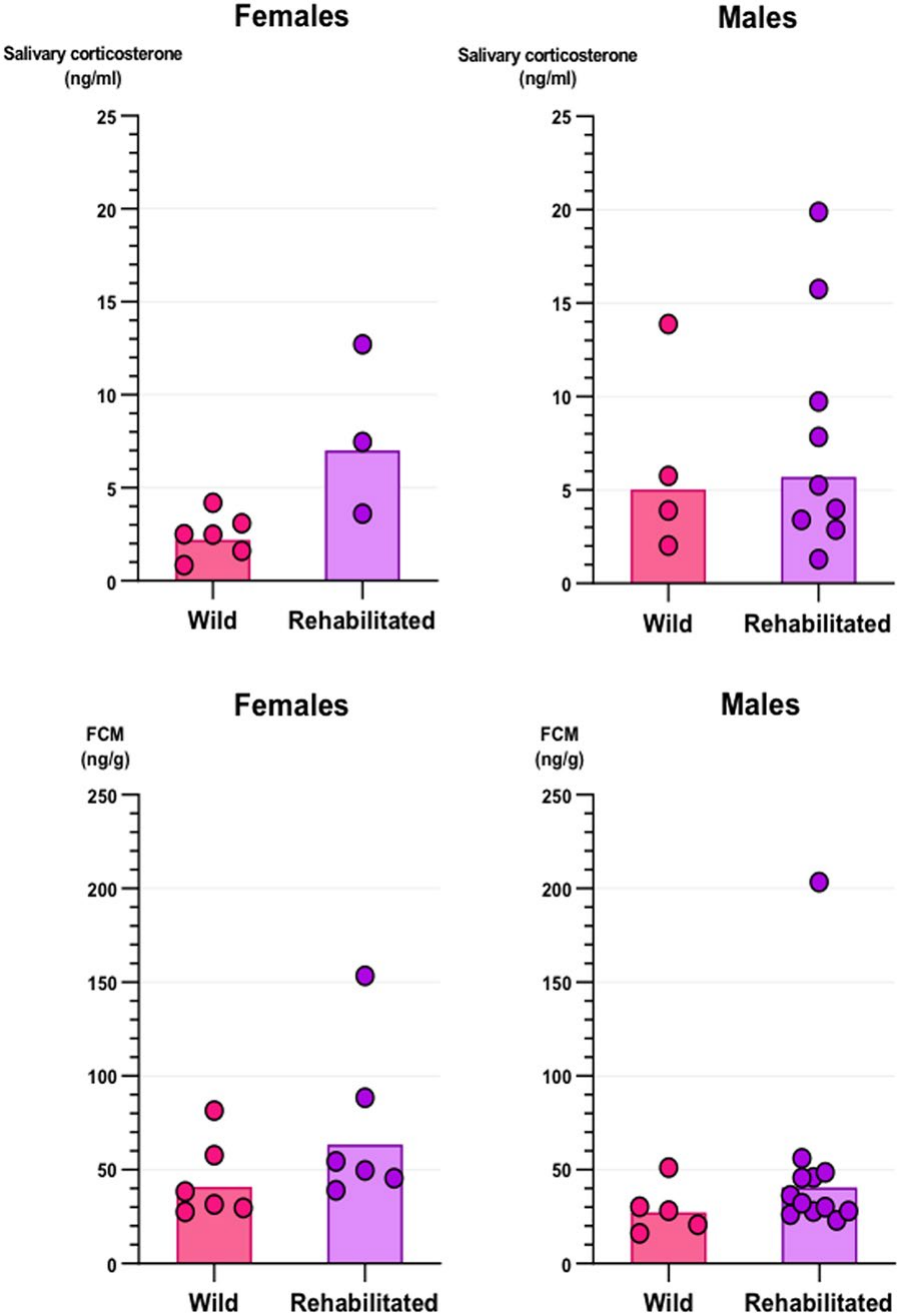
Salivary corticosterone and faecal corticosterone metabolite (FCM) levels in wild and rehabilitated individuals of both sexes. Markers denote individuals. Where multiple samples were analysed, an average is presented for the single individual. Bars represent the geometric mean for each group, as glucocorticoid data is known to conform to log-normal distributions

The measured saliva corticosterone levels from individuals in cohort 2 ranged between 0.41 and 59.96 (mean = 7.69 ± 9.83 ng/mL, n = 57). Only the background (wild/rehabilitated) of the subjects appeared to have an effect on saliva corticosterone levels in the present study (χ^2^(1) = 5.58, p = 0.018, n = 55), with wild individuals having significantly lower levels of saliva corticosterone compared to rehabilitated individuals (Fig. 1).

### Faecal corticosterone metabolite levels

The detected faecal corticosterone metabolite levels ranged between 15.33 and 369.5 ng/g (n = 86). However, only 43 samples from 29 individuals with representation from all three cohorts were included in the data analysis, as the remaining samples were collected from enclosures with more than one individual (cohort 2) or randomly in the wild (cohort 3), and could therefore not be allocated to a specific individual, which was a necessary information for the type of data analysis chosen. The faecal corticosterone metabolite levels for samples included in the data analysis still ranged between 15.33 and 369.5 ng/g (n = 43) with a mean of 53.3 ± 58.2 ng/g.

We failed to find an effect of health status (dying from *Salmonella* or not) on faecal corticosterone metabolite (FCM) concentrations. However, both sex and background appeared to influence FCM levels: rehabilitated hedgehogs had significantly higher FCM levels than wild hedgehogs, and females had significantly higher levels than males in the present study (χ^2^(1) = 6.98, p = 0.008).

### Cortisol levels

Faecal cortisol metabolite levels of 19.85–79.30 ng/g, with a mean of 41.29 ± 21.67 ng/g, were detected in 7 faecal samples from six different individuals from cohort 3. Cortisol levels of 2.16–15.34 ng/mL, with a mean of 10.14 ± 6.15 ng/mL, were measured in four saliva samples from individuals belonging to cohort 3. Due to the low sample size, we refrained from further data analysis.

### Novel arena test

The data obtained in the novel arena test was condensed using PCA. Two components, explaining 84% of the variance in data, were extracted, based on scree plot analysis. Moreover, due to the small sample size in relation to the number of dependent variables, extracting more than two latent trends was deemed excessive. The two components were tentatively interpreted as a measure of fearfulness (shyness/boldness) (PC1) and general activity level (PC2), respectively, based on their factor loadings (Additional file 5). PC1 correlated strongly (positively) with the time individuals spent lingering in, or near, the carrier, but also correlated strongly (negatively) with the time spent in the distant zones. This suggests a shy/bold axis. The second component correlated (positively) with the total number of zone transitions and frequency of entries into the distant zones. This suggests an axis of general activity level. No clear trends could be found with respect to any of the two latent trends on a group level. Neither sex, health status, nor background appeared to have an effect on either boldness or general activity as trends were investigated using analysis of variance.

### Novel object tests

Similar to the novel arena test, data were subjected to condensation using PCA and two components were extracted based on scree plot analysis, explaining 88% of the variance in the dataset. The two components split neatly between the two tests, PC1 describing the fearfulness shown in the ball test and PC2 describing the fearfulness in the badger test (Additional file 4). This suggests that the responses in the two tests were somewhat independent of one another.

Whereas we expected to see an effect of testing order caused by habituation (whether individuals were subjected to the ball or badger setup for the first novel arena test), this was not evident from the limited data (Additional file 6). Consequently, testing order was excluded as an explanatory variable in further testing. On a group level, two trends could be discerned. Subjects with a “rehabilitated” background appeared to present with a higher average PC2 score (F_1,14_ = 5.49, p = 0.034), suggesting a more timid behavioural response in the badger test. Sick individuals presented with a slightly lower PC1 score (F_1,14_ = 4.68, p = 0.048), suggesting a less timid response in the ball test (Additional file 7).

### Personality

A total of 24 individuals from cohort 2 were tested for personality measured as shyness-boldness. 13 individuals were labelled as shy and 11 individuals as bold. The distribution of shy and bold individuals based on background were five shy and five bold for wild individuals; eight shy and six bold for hand-reared, rehabilitated individuals. See Additional files 8, 9, 10 and 11 for personality test results and an overview of the distribution of shyness and boldness per individual.

### Post release survival, personality and hibernation behaviour of cohort 2

Post release survival from release during the autumn and until initiation of hibernation was 53% (n = 9 out of 17). A total of four out of seven hand-reared, rehabilitated individuals survived (57%) and five out of ten wild individuals survived (50%). Originally, eight hand-reared, rehabilitated individuals were released, but the radio signal was lost from one, which was consequently excluded from the survival analyses. Causes of death were predation by badgers (n = 3, two wild, one rehabilitated individual), *Salmonella* infections (n = 4, two wild, two rehabilitated), and one wild individual was stepped on by a cow. The difference between post release survival rates of wild and hand-raised individuals was not statistically significant (Fisher’s Exact Test, two-tailed P value = 1.00). Personality, measured as shyness–boldness, did not influence post release survival in the present study (Fisher’s Exact Test, two-tailed P value = 1.00), with 4 shy individuals dying, 5 shy individuals surviving, 4 bold individuals dying and 4 bold individuals surviving, post release, until hibernation.

Individuals dying post release (n = 8) did so within 9 days after release (range 2–9 days). Hibernation was initiated between 31st of October and 17th of November, the majority (n = 6) around mid-November. The hedgehogs began hibernating 6–38 days post release (n = 9) depending on the release date, as individuals released late in the season initiated hibernation quite promptly.

### Post release spatial behaviour of individuals from cohort 2

Based on the GPS coordinates obtained from the post release radio tracking, home range estimates were made for individuals W7 (n = 35) and W8 (n = 28) in cohort 2, being the only individuals with sufficient data points (> 30) for calculating representative home range estimates [75]. 95% minimum convex polygons: W7 (4.85 ha) and W8 (3.54 ha). Kernel density estimates: W7 (95%: 7.07 ha, 50%: 0.12 ha) and W8 (95%: 5.58 ha, 50%: 0.06 ha),

For comparison, home ranges for individuals from cohort 3 can be found in Rasmussen et al. (2019) [22]. Mean dispersal length from the release sites was 217 ± 100 m (range 100–408 m), measured as the greatest distance from the release sites recorded per individual, for 11 individuals in cohort 2 released back into the wild. The dispersal lengths were measured during the period post release until initiation of hibernation, ranging from 6 to 38 days. The remaining six individuals (range 5–45 m) were excluded from the analysis because they died shortly after release and never got to explore the new habitats. Dispersal length was equal for rehabilitated (212 ± 102 m, range: 100–322, n = 4) and wild individuals (219 ± 106 m, range: 101–408, n = 7).

## Discussion

During our tests of glucocorticoids in hedgehogs we discovered that rehabilitated individuals and females had higher levels of faecal corticosterone metabolites compared to wild individuals and males, respectively. Furthermore, rehabilitated individuals showed higher levels of saliva corticosterone than wild.

The difference detected in faecal corticosterone metabolite levels between males and females is most likely a general sex difference, which has previously been detected in a range of species (e.g., [76–80]) and was previously hinted at by Fowler (1988) [81].

Rehabilitated individuals had significantly higher levels of corticosterone and corticosterone metabolites in both saliva and faeces, respectively, compared to the wild individuals in the study, and the high occurrence of *Salmonella* infections (category labelled as “health” in the statistical models) among the rehabilitated individuals did not affect the results. Taking into consideration that ten of the wild individuals included in the analyses were kept in captivity for a week under the same conditions as 14 of the rehabilitated individuals tested (cohort 2), the results could indicate that the rehabilitated individuals were in general more stressed, having been kept in captivity for a longer period of time and having been moved from one enclosure to another. It is therefore relevant to consider the length of the rehabilitation process and the potential negative consequences of a long admission to a wildlife rehabilitation centre, as well as whether the benefits of moving individuals to new enclosures for a soft release will outweigh the potential increase in stress levels caused by this act. However, further studies on the effects of translocation to new enclosures are needed to confirm this.

Previous studies have investigated different aspects of adrenal function and adrenal hormone levels in hedgehogs [81–88]. These studies have principally focused on glucocorticoid-involvement in relation to/in preparation for hibernation. Both corticosterone [84] and cortisol [81] have been measured in this context in hedgehogs, however, to our knowledge, there are no studies of either glucocorticoid’s involvement in stress. Similar to, for example, hamsters [89], hedgehogs appear to secrete considerable levels of both cortisol and corticosterone (as opposed to most mammalian species where there is a considerable skew toward one of the two). Comparing the faecal samples from cohort 3, where we could obtain reliable measurements of both faecal corticosterone- and cortisol metabolite levels, we found the average level of corticosterone metabolites (48.4 ± 24.92 ng/g) to be higher than that of cortisol metabolites (41.3 ± 21.7 ng/g). However, the mean saliva cortisol level in general (10.1 ± 6.1 ng/ml) was higher than that of corticosterone (7.7 ± 9.8 ng/ml) detected in the saliva samples. This supports the findings by Werner and Wünnenberg (1980) [88] who found 3–4 times higher levels of cortisol in hedgehog plasma. It is, therefore, tempting to suggest that cortisol should be focused on as the primary stress-associated glucocorticoid. But we would argue that this would be a premature conclusion since it has been suggested that cortisol and corticosterone may take on different roles [90] in species where both hormones are found in appreciable concentrations (e.g., hamsters [91] or bats [92]). To move forward, there is, instead, a need for validating cortisol and corticosterone concentrations in European hedgehogs in relation to controlled stressors; for example, ACTH challenges. As the European hedgehog is protected by law in Denmark, such experiments would require specific permits which are not easily obtainable.

Unfortunately, we had to exclude a number of faecal samples (n = 43) from the data analysis of faecal corticosterone metabolite levels, because they could not be assigned to a specific individual.

Seven out of 15 rehabilitated individuals from cohort 2 died from *Salmonella* infections before release back into the wild. Four individuals (two wild and two rehabilitated) died of *Salmonella* infections post release, showing no symptoms before release. One could anticipate that sick individuals would not behave as they would have done under normal conditions, which could influence the results of the personality tests. Surprisingly, we observed that individuals dying from *Salmonella* infections were among the most active (bold) individuals during the personality tests, even though apathetic behaviour could have been expected. The detected levels of corticosterone may also have been affected by disease, but this could not be confirmed by the statistical analyses, as health status did not significantly influence corticosterone levels. However, the *Salmonella* infections did reduce our post release sample size (cohort 2) considerably, which should be taken into consideration when interpreting the results.

The majority of personality studies have been carried out on captive-bred individuals [93]. Archard and Braithwaite [93] stated that there is a need for personality tests of wild populations in order to discover the selection pressures that affect personality in natural environments. The stress associated with the capture, handling and captivity of wildlife should be considered [93] as well as a potential bias in trapping wild animals for research, since “trappability” of wild animals has been used as a measure of boldness in previous studies [59, 94]. However, five out of ten wild individuals were categorised as bold in the present study, which does not indicate any “trappability” bias. Yet, given the small sample size, it could also just be caused by coincidence. Additionally, it is relevant to mention that some individuals may have personalities that allow them to thrive in rehabilitation, which could give them an advantage upon release.

Fucikova et al. [95] describe how handling stress can potentially influence the behaviour in personality tests, and how this should be taken into consideration when interpreting the results. As the rehabilitated, hand-raised individuals of cohort 2 should be more habituated to handling by humans, the wild individuals would then be expected to show more shy behaviour compared to the rehabilitated individuals in the personality test, if they were influenced by handling stress. This was not the case, as only five out of ten wild individuals were labelled as shy, compared to eight out of 14 rehabilitated individuals. However, some individuals may also appear to be bold whilst masking very high stress levels [96]. This did not seem to be the case in the present study, as personality did not affect stress levels.

Post release survival did not appear to be affected by background or personality in the present study. However, it is important to consider the potential biases caused by the rather small sample size (n = 17). All individuals dying post release did so within 9 days after release, which is remarkable, and may indicate that if an individual is able to survive after approximately the first week post release, there is a good chance that it will survive until hibernation. Post release survival of rehabilitated hedgehogs has previously been found to range between 25 and 83% [30, 67–73]. Yarnell et al. (2019) [73] detected no significant difference between the survival of wild and rehabilitated hedgehogs (n = 42, overall survival rate = 83%) during the first 150 days after release of the rehabilitated individuals. Rasmussen et al. (2019) [22] found a survival rate of 78% (n = 23) for wild, juvenile hedgehogs during the autumn until initiation of hibernation. The post release survival rate of 53% observed in the present study is remarkably lower and is likely due to the presence of the *Salmonella* infection, which killed four individuals post release. However, the survival rate of 53% in the present study is still average compared to the range of 25–83% found in previous studies of post release survival of rehabilitated individuals.

The post release home range sizes measured for individuals of cohort 2 were almost equal to the levels found in Rasmussen et al. (2019) [22], where wild juvenile hedgehogs were radio tracked in the same area as used in the present study. Combined with the small post release dispersal length of 217 ± 100 m we detected, especially compared to previous studies on translocated hedgehogs [30, 67, 69, 71], it seems translocated, juvenile hedgehogs do not travel far from their release site and stay in a small area, if the habitat is of suitable quality. This suggests that future post release monitoring of hand-raised orphans should be possible for hedgehog carers even without the use of tracking equipment.

## Conclusions

In conclusion, we determined the personality measured as shyness-boldness of 24 independent, juvenile hedgehogs with different backgrounds. Afterwards, they were radio tagged and released into a novel habitat. We found no difference in the post release survival of hand-reared rehabilitated and wild, juvenile European hedgehogs, and survival did not seem to be affected by personality. These results show that hand-raised, rehabilitated juveniles have the same prospects post release as wild individuals brought up naturally, and that chances of post release survival are seemingly not influenced by personality (shyness-boldness).

We measured glucocorticoid levels in 43 European hedgehogs from different backgrounds, age groups and locations using commercially available assays for corticosterone and cortisol to quantitate (an unknown mix of) the native glucocorticoids and their metabolites. We found that rehabilitated individuals had higher levels of corticosterone metabolites (faeces) and corticosterone (saliva) compared to wild individuals. Additionally, females had higher levels of saliva corticosterone than males, but this was most likely a general sex difference. The results indicate that rehabilitated individuals show higher levels of saliva corticosterone and faecal corticosterone metabolites than wild individuals, likely due to a longer stay in captivity. Based on these observations we suggest that the duration of admission to hedgehog rehabilitation centres should be considered. However, more research on the subject is needed, particularly a validation of the detected levels of cortisol and corticosterone in European hedgehogs through ACTH tests, before we can draw any definitive conclusions on the stress levels of the individuals studied.

## Methods

The samples in the study came from 43 individual hedgehogs (25 rehabilitated and 18 wild) collected over the course of three different research projects. In addition to sex, individuals were defined either as wild or rehabilitated (ten sick/injured adults being treated at a wildlife rehabilitation centre (cohort 1) and 15 hand-reared orphans (part of cohort 2)). Table 1 provides a flow chart of the entire research setup.

**Table 1:** A flow chart presenting the research setup for the three cohorts studied

Categories		Cohort 1		Cohort 2		Cohort 3
Subject characteristics		n = 10 rehabilitated, adult hedgehogs		n = 15 rehabilitated, juvenile hedgehogs		n = 8 wild hedgehogs (3 juveniles, 5 unidentified)
				n = 10 wild, juvenile hedgehogs		
Glucocorticoid analyses						
Sampling		Faecal samples (n = 10)		Saliva samples (n = 57)		Saliva samples (n = 4)
				Faecal samples (n = 67)		Faecal samples (n = 9)
				*Sampling before (wild) and during captivity (both groups) and post release (both groups)*		
Laboratory procedure		*Testing:*		*Testing:*		*Testing:*
		Faecal corticosterone metabolite levels (n = 10)		Faecal corticosterone metabolite levels (n = 67)		Faecal corticosterone metabolite levels (n = 9)
				Saliva corticosterone levels (n = 57)		Faecal cortisol metabolite levels (n = 7)
						Saliva cortisol levels (n = 4)
Personality testing		Not performed		Novel arena test on day 1 in enclosures		Not performed
				Novel object tests on day 3 and 5 in enclosures		
Release into the wild		Not performed		After 7 days in enclosures (n = 18, 8 rehabilitated and 10 wild juveniles)		Already free-living
Post release monitoring of survival and spatial behaviour		Not performed		Radio tracking of 18 juveniles, out of which 1 was unaccounted for		Results not included in the present study: Radio tracking of the 3 radio tagged wild juveniles (Rasmussen et al. (2019))
Data analyses						
Faecal corticosterone metabolite levels in relation to background, sex and health		Linear mixed effects model (LME) for all cohorts		Linear mixed effects model (LME) for all cohorts		Linear mixed effects model (LME) for all cohorts
Saliva corticosterone levels in relation to background, sex and health				Linear mixed effects model (LME)		
Personality (novel arena and novel object tests)				Principal component analyses (PCA)		
Effects of personality (shy/bold) and background (wild/rehabilitated) on post release survival				Fisher's exact test		

### Subject characteristics

#### Cohort 1

Cohort 1 consisted of ten adult hedgehogs in care due to either injuries or sickness at a hedgehog rehabilitation centre near Copenhagen. Samples, one saliva and one faecal sample per individual, were collected on the 2nd of July 2012. See Additional file 1 for information on weight, sex and conditions of the individuals.

#### Cohort 2

Cohort 2 consisted of ten wild juvenile hedgehogs (estimated age > 6 weeks) and 15 hand-reared orphans (7–8 weeks old). All were from Zealand, Denmark; born between July and the beginning of September 2012. See Additional file 2 for further information. The orphans were resident at two wildlife rehabilitation centres (operating under Dyrenes Beskyttelse) for at least 3 weeks before entering the study. All animals were over the age of independence [1, 97]. The wild individuals were hand caught in the suburbs of Copenhagen using headlights and night vision goggles. Wild-caught and hand-reared hedgehogs were separately housed in outdoor enclosures under the same conditions to facilitate direct comparisons between the two groups. Each chicken-wire enclosure (3 m × 2 m) had a chipboard roof and housed 2 individuals. Nest boxes (50 cm × 50 cm × 40 cm) were provided with sawdust on a sheet of surgical base, hay, a bowl of water and a bowl of kitten dry food. Additional kitten wet food placed beside the box entrance. Food and water was also present in the pen. Food and water were changed daily, and the nest boxes were cleaned thoroughly every second to 3rd day.

##### Experimental design for cohort 2

Each individual was colour coded for identification purposes using Hama beads (www.hamabeads.com) glued to its spines. Hedgehogs were weighed and faecal samples collected daily. On day 1 (arrival), the hedgehogs were tested in a novel arena setting. On days 3 and 5 they were exposed to the novel object test. On day 6, radio transmitters were attached, and the individuals were released at night on day 7.

#### Cohort 3

This group consisted of four radio-tagged wild, juvenile hedgehogs, aged approximately 8 months at the time when the faeces and saliva samples were obtained. A more detailed description of the individuals and the study in which they participated can be found in Rasmussen et al. (2019) [22]. Faecal samples from five wild, unidentified individuals from Taastrup and Rødovre were also included to increase the representation of wild individuals in the study. All samples were collected in May 2015.

### Sampling methods

Faecal samples were collected and frozen (− 20 °C) as soon as possible, at the latest within 30 min after the samples were collected, awaiting analysis.

The saliva samples were collected with a single-use pipette (model LW4273 Alphalabs UK), kept in cooler bags directly after sampling, and stored frozen (− 20 °C). The saliva samples were collected by gently placing the long and narrow tip of the single-use pipette in the corner of the mouth of the hedgehog. Saliva was aspirated from the inside of the individual’s cheek, after which the pipette was gently extracted from the mouth of the hedgehog. During the procedure, the hedgehog was placed in the hands of the person extracting the sample and was not restrained. Each procedure lasted < 20 s. The saliva sampling took place at the first given opportunity during their activity period at night, when handling the hedgehogs, in an attempt to avoid detecting the stress from handling in the samples. The first saliva sample for individuals belonging to cohort 2 was taken upon arrival to the pens, 15 min before the novel arena test. In some instances further samples were obtained before the novel object tests. The last samples were collected upon tagging and release into the wild, and in some cases, when it was possible to catch the individual, post release.

### Laboratory procedure

A total of 57 saliva samples and 86 faecal samples were analysed for glucocorticoids. Corticosterone and corticosterone metabolites in saliva, as well as faecal corticosterone metabolites (FCM) were quantitated using a commercially available corticosterone assay. Similarly, faecal cortisol metabolite levels were assessed in seven faecal samples and an additional four saliva samples were analysed for cortisol levels using a commercially available cortisol assay. Saliva samples (n = 61) were analysed neat or diluted in PBS where needed. Faecal samples (n = 93) were extracted in ethanol (96%) overnight. The supernatant was recovered by centrifugation, evaporated, and the extracted material was resuspended in PBS prior to analysis. Both cortisol and corticosterone concentrations were measured in the samples, but due to a highly pronounced matrix effect in the initially chosen assay for cortisol quantification (“Cortisol ELISA”, EIA-1887; DRG Instruments GmbH, Germany), combined with limited sample material (faeces and saliva samples from cohort 1), the results from a number of cortisol analyses had to be discarded. Corticosterone concentrations were determined using commercially available ELISA kits (“Corticosterone ELISA”, REF EIA-4164; DRG Instruments GmbH, Germany). Known cross-reactivities are with progesterone (7.4%), deoxycorticosterone (3.4%), 11-deoxycorticosterone (1.6%), cortisol (0.3%), and pregnenolone (0.3%), with other steroids cross-reacting at less than 0.1%. Sensitivity of the kit (detection limit) is listed at 1.6 nM and typical intra- and inter-assay CVs are listed at 3% and 6%, respectively. A small subset of saliva (n = 4 from cohort 3) and faecal (n = 9 from cohort 3) samples that had not been exhausted were analysed for cortisol concentrations using an assay which was deemed reliable, and did not show signs of matrix effects (“Parameter cortisol assay”, KGE008, R&D Systems Parameter Cortisol). Known cross-reactivities are with prednisolone (4.4%), 11-deoxycortisol (3.4%), progesterone (1.7%), and cortisone (0.2%), with other steroids, including corticosterone cross-reacting at less than 0.1%. Sensitivity (detection limit) of the kit is listed at 0.071 ng/ml and typical intra- and inter-assay CVs are listed at 5% and 9%, respectively.

Despite the need for validating cortisol and corticosterone concentrations in European hedgehogs in relation to controlled stressors with for example ACTH challenges [98], it was unfortunately not possible to provide such a validation in the present study, as the European hedgehog is protected by law in Denmark, and such experiments would require specific permits which are not easily obtainable.

### Personality testing

Individuals from cohort 2 were tested in the novel arena test and the novel object test before being released into the wild.

### Novel arena test

**Fig. 2:**
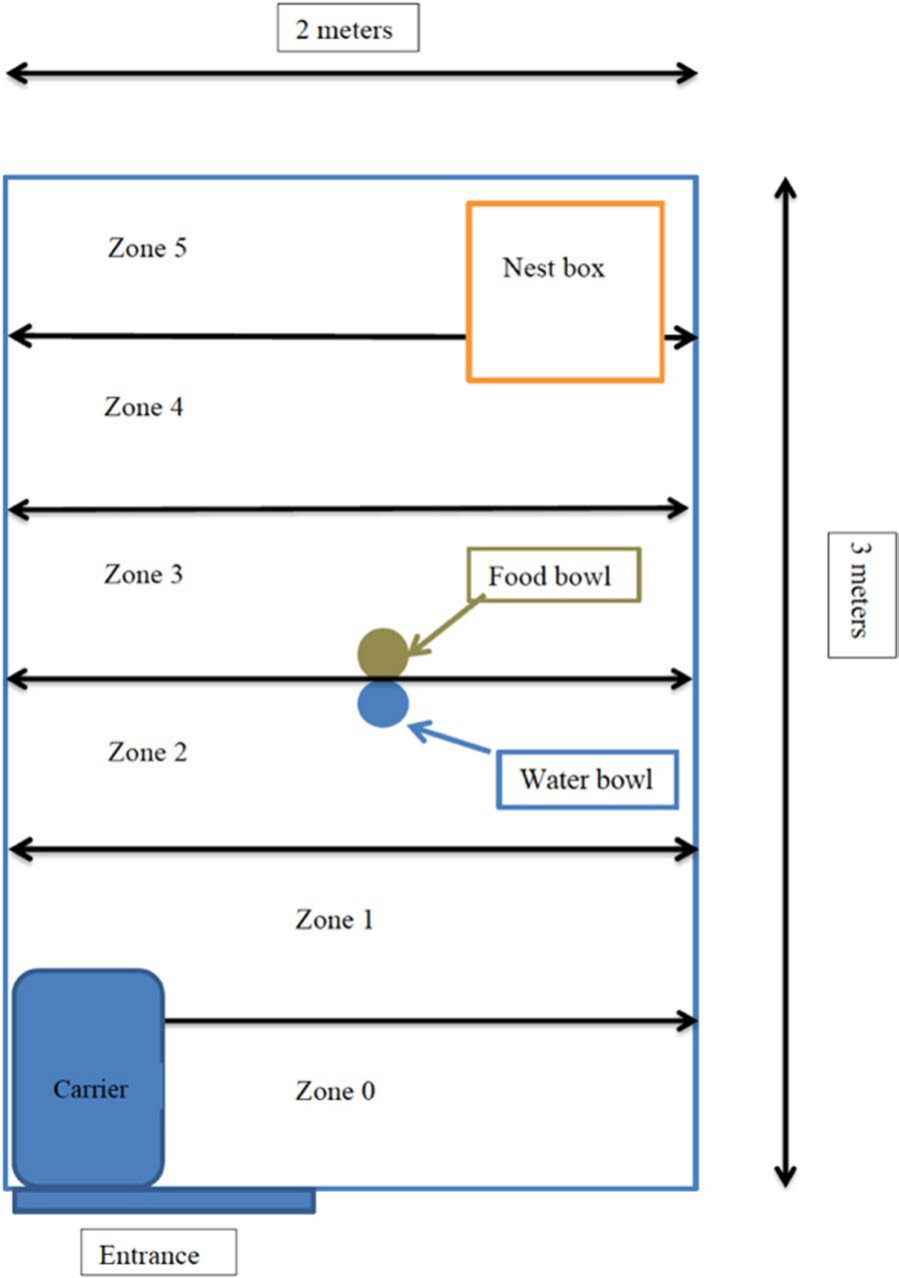
The novel arena test setup. The test was made when the individual entered the arena (enclosure) for the first time, on day 1. The arena was divided into zones. The individual was placed in the carrier in zone 0, and the carrier was opened when the test started. Test duration was 15 min. A researcher monitored the events from outside the enclosure and recorded the test results

When released into the enclosure for the first time, the hedgehogs were tested in a novel arena paradigm. The enclosures were divided, lengthwise, into zones of 50 cm (numbered 0–5) by use of strings which were woven into the chicken-wire netting placed in the ground-level of the enclosures. The nest box was placed in zone 5 extending into zone 4, but was kept shut during the test. The outdoor water and food bowls were placed in zone 3 (Fig. 2). Each individual was brought to the arena in a cat transport carrier with bedding and left near the enclosure for 15 min prior to the experiment. The transport box was gently placed in the entrance corner of the enclosure (zone 0) and opened. During the next 15 min, the hedgehog’s latency to exit the carrier (t_out_), latency to enter the different zones (t_1–5_) and the total time spent in each zone (Σ_0_–Σ_5_), was recorded, as well as the total number of visits to each zone (visits_1–5_) and the total number of borders crossed (total visits). Entering a zone was defined as having moved the entire body into the zone. The recording of the novel arena tests was made by the same, single observer, with the exception of two occasions, where a second person was also present alongside the observer. The observer wore a headlight pointing downwards to light up the registration sheet, and made the observations from a distance of 2 m from the enclosure staying silently in place for the duration of the experiment. The transport box was cleaned between each test.

### Novel object tests

**Fig. 3:**
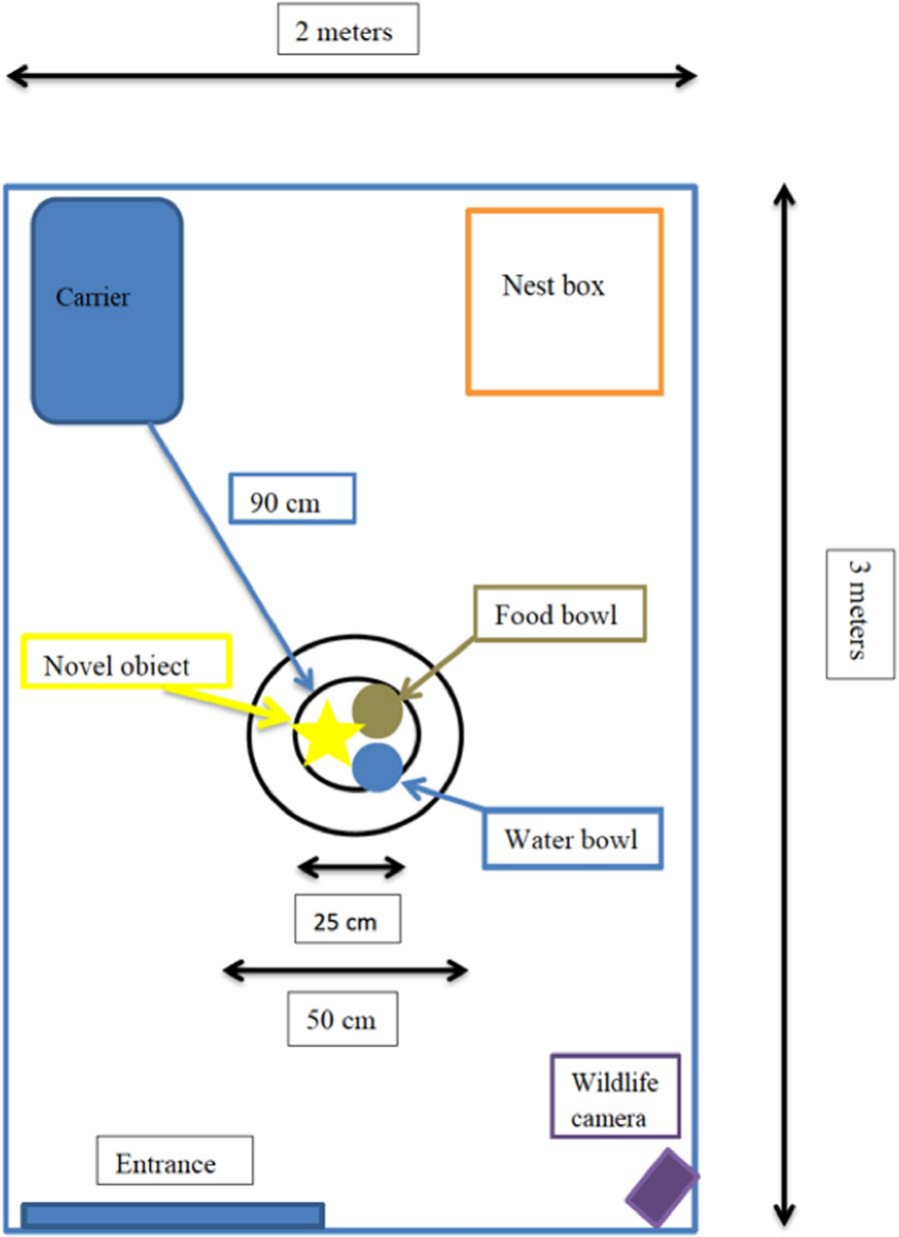
The novel object test setup. Individuals were tested in the novel object test setup on day 3 and 5 in the enclosure. The novel objects were a ball and a badger setup. Trying to avoid habituation bias, some individuals were tested with the ball as the first novel object test, and some were tested with the badger as the first novel object test. Test duration was 90 min. A wildlife camera in the lower right corner of the enclosure recorded the test situations

On day 3 and day 5 the response of the hedgehogs in cohort 2 to a novel object was tested. The objects were a pink football (25 cm in diameter) and a badger setup consisting of a small paper box with badger faeces and a stuffed animal toy with white and black colours, mimicking a badger pup (around 40 cm in length). New samples of badger faeces were provided for each test night. The badger setup was chosen as badgers are natural predators of hedgehogs [15, 97, 99] and the football was chosen as a neutral item, with the two novel object tests reflecting personality in a relatively neutral and a more threatening environment [100, 101]. The novel items were placed in the centre of the enclosure next to the outdoor food and water bowls. The hedgehog was gently placed in an open carrier next to the closed nest box at a distance of 90 cm from the novel object (Fig. 3). Each test was filmed with a Prostalk PC3000IR wildlife trail camera, which was set to record for 90 s after each detected movement in the enclosure, with a time lag of two minutes between recordings. The placement of the wildlife camera in the right corner of the enclosure ensured a full view of the arena and therefore accurate measurements of distances. All novel object tests lasted 90 min and were carried out at night (the active period of hedgehogs). Each hedgehog was tested individually, whilst the other individual housed in the same enclosure was confined to the nest box. The latency to exit the carrier (t_out_), the latency to approach the novel object, t_app_ (defined as coming within 50 cm of the object), and the smallest distance from the object were recorded. The distance from the object was measured to the tip of the hedgehog’s snout. All videos were coded by a single observer.

The testing order (ball/badger) was randomized and took place on day 3 and 5. Each testing session ideally consisted of two wild and two rehabilitated individuals in separate enclosures, one housing the wild individuals and the other the rehabilitated. With a few exceptions, each individual was tested once in each of the two setups. 21 individuals were tested in the ball setup and 18 in the badger setup (the uneven number of tests was caused by deaths among the test individuals).

### Salmonella detection

Seven out of 15 hand-raised, rehabilitated individuals from cohort 2 died from *Salmonella* infections before release back into the wild. They had contracted the infections during care at a wildlife rehabilitation centre due to the lack of necessary hygienic precautions and diagnosis/treatment. Four individuals from cohort 2 (two wild and two rehabilitated) died of *Salmonella* infections post release, showing no symptoms before release. All cases were confirmed through PCR validation and categorised as causes of death during the necropsies conducted at Wildlifehealth.dk.

### Spatial behaviour and survival post release

After staying in the enclosures for 7 days, the surviving hedgehogs of cohort 2 (eight hand-raised and ten wild individuals) were released back into the wild wearing radio transmitters (Biotrack PIP transmitters of 3–4 g). The purpose was to compare the post release success of wild and hand-raised, rehabilitated individuals. A total of seven groups, each consisting of both hand-raised and wild individuals, were released at six different locations between 14th of September and 8th of November 2012, after spending 6 days in the enclosures during the personality testing (Additional file 2).

The hedgehogs were transported to the release site in two carriers, wild and rehabilitated housed separately. The hedgehogs were allowed to exit the carriers at their own speed. Dry cat food and water was offered for a week post release, and the two carriers were not removed until a week post release, providing the individuals with an alternative nest site during the first week after translocation into the foreign area. The first two releases were made in the area of Gribskov in a forest edge habitat surrounded by grassland and containing a single house and garden which was evaluated as suitable based on a previous study on the habitat types of Danish hedgehogs [102] (Latitude, longitude: 55.975983, 12.266367; 55.974898, 12.264987). Afterwards new release sites were found in Taastrup in a large recreational area suitable for release due to the presence of a large hedgehog population, adjacent to residential areas, and with the presence of foxes (Latitude, longitude: 55.642388, 12.328723; 55.643445, 12.331849; 55.649443, 12.333854; 55.651115, 12.328648). The change of release site was prompted by a surprisingly high predator (badger and fox) density in Gribskov, likely causing multiple deaths (n = 3) among the hedgehogs during the first days post release. All wild-caught hedgehogs were released at least 3 km from their capture site to avoid a possible bias of advantages due to acquaintance with local conditions. The measure of 3 km was based on previous studies of adult hedgehogs, wherein the largest observed distance travelled in a night was 2 km, and adult home ranges were < 40 ha [1].

Post release, the hedgehogs were radio tracked with a Sika receiver and Yagi antenna and found every one-two nights post release. Their locations were recorded with a Garmin eTrex 20 GPS. The radio tracking was carried out in the activity periods of the hedgehogs, between 8 p.m. and 3 a.m., in order to cover the two possible peaks of activity, 21:00–24:00 and around 3:00 o’clock, as suggested by Campbell [103] and Wroot [104]. Only one position was recorded each night (in different hours of the night) as an attempt to obtain independent data for the calculation of home ranges [105]. The surviving hedgehogs were followed until initiation of hibernation. Post release survival and home ranges were measured and compared between treatment groups (wild and rehabilitated juveniles). A total of 18 individuals were radio tagged and released back into the wild, but the signal was lost from one rehabilitated individual shortly after release, which means we excluded this individual from the different post release data analyses.

### Data analysis

All measures of dispersion in the manuscript are listed as standard deviation (SD).

The linear mixed effects models (LME) were prepared and tested using the software R [106]. The principal component analyses were performed in SPSS v. 25 [107].

### Saliva corticosterone samples

A linear mixed effects model (LME) was used for analysing the corticosterone levels found in saliva. The model included the subjects as a random effect as some individuals contributed with multiple saliva samples. The corticosterone levels were log-transformed prior to analysis to obtain a normal distribution (Shapiro–Wilk normality test, post transformation: W = 0.9893, p = 0.9051). An initial model included the background (wild/rehabilitated), sex and health (dying from *Salmonella* infection or not) as fixed effects.

Stepwise reduction of the model’s explanatory values was subsequently employed, using ANOVA tests to compare the models, gradually removing non-significant terms from the models indicated by the results of the ANOVA tests. The best fit model only included the background of the subjects in addition to the random effect of subject identity (lmer (log-corticosterone ~ Background + (1|Individual), data = Corticosterone_Data).

### Faecal corticosterone metabolite levels

We prepared a linear mixed effects model (LME) for the statistical analyses of the faecal corticosterone metabolite levels detected. The model included the subjects as a random effect as some individuals contributed with multiple samples. The faecal corticosterone metabolite levels were log-transformed prior to analysis to obtain normal distribution. An initial model included the background (wild/rehabilitated), sex and health (dying from *Salmonella* infection or not) as fixed effects. As the data was analysed with faecal corticosterone metabolite levels per individual as the response variable, we had to exclude a number of faecal samples (n = 43) collected from enclosures with more than one individual (cohort 2), or from unknown wild individuals (cohort 3), unless we knew exactly which individual the sample came from.

Stepwise reduction of the model’s explanatory values was subsequently employed, using ANOVA tests to compare the models, gradually removing non-significant terms from the models indicated by the results of the ANOVA tests. The best fit model included the background of the subjects, and sex, in addition to the random effect of subject identity (lmer (log-corticosterone ~ Background + Sex + (1|Individual), data = Corticosterone_Data).

### Personality tests

To facilitate analysis/interpretation, the data collected from both the novel arena tests and the novel object tests were dimensionally reduced using principal component analysis (PCA). One analysis utilized the data collected from the novel arena test, a separate analysis was carried out for the novel object test (combining data from both testing conditions—ball/badger). Varimax rotation was employed, post-extraction, to facilitate easier interpretation of data. Whereas the number of parameters used in both PCAs was high in relation to the number of subjects, this approach was still deemed preferable over analysing the parameters individually in, for example, ANOVA models.

### The effects of personality and background on survival

Shyness or boldness was established for each of 24 individuals in cohort 2 based on the three personality tests (novel arena, novel object ball, novel object badger). Each individual therefore received three shyness/boldness labels and were registered as either shy or bold on the basis of these. In cases where both shyness and boldness labels were allocated to the same individual, the majority determined the final personality label (e.g. shy, bold, shy was labelled shy). Due to challenges during the initiation phase of the personality tests, two of the individuals surviving to be released back into the wild did not take part in the novel object tests, and were consequently labelled shy or bold based on their performance in the novel arena test. A further four rehabilitated individuals who did not survive to be released, were additionally categorised as shy or bold based on their results from the novel arena test. The division of individuals into a shyness-boldness spectrum was based on the clustering in the PCA space by visually distinguishing and ordering the individuals into the binary categories of shy and bold (See Additional files 3 and 4). Half of the individuals were categorised as shy and half as bold based on each of the three PCs representing the tests (PCA plot for novel arena = PC1, n = 24; PCA plot for novel object tests: PC1 and PC2, n = 18).

Fisher’s Exact test was employed to test for an effect of background (wild/rehabilitated) and personality (shy/bold) in the post release survival of the 17 released individuals from cohort 2.

### Post release spatial behaviour of individuals from cohort 2

The calculations of home ranges, measured as minimum convex polygons, and kernel density estimates were made in ArcGIS 10.0 by application of the extension program Geospatial Modelling Environment. For the kernel density calculations, bandwidth was set to 500 m and cell size to 1 m, as these settings created the smoothest kernels. We measured the maximum distance from the release point in Google Maps for the 17 individuals surviving to be released back into the wild.

## Supplementary Information

**Additional file 1.** Overview of individuals from cohort 1. The individuals of cohort 1 were all in care at a local hedgehog rehabilitation centre.

**Additional file 2.** Overview of individuals in cohort 2. The column weight indicates the weight in grams of an individual when entering the study.

**Additional file 3.** Novel arena tests: Distribution of individuals in PCA space. Note that the labelling of the axes is speculative.

**Additional file 4.** Novel object tests: Distribution of individuals in PCA space. Note that the labelling of the axes is speculative.

**Additional file 5.** PC scores for the novel arena test data.

**Additional file 6.** Novel object tests: Distribution of subjects in PCA space, labelled by order of test. No obvious effect of testing order can be seen.

**Additional file 7.** PC scores for the novel object tests’ data.

**Additional file 8.** Personality. A table presenting the division of individuals into shy or bold based on their behaviour in the three personality tests. Shy behaviour is indicated by S and bold by B.

**Additional file 9.** Results from the novel object test with a badger setup. A table presenting the results from the novel object test with the badger. Total duration: 90 min. “Type” indicates whether the individual was tested in the novel object test scenario with the badger as the first test (NO1) or the second test (NO2). ∆t out is the latency time before the individual left the carrier and entered the arena.

**Additional file 10.** Results from the novel object test with a ball setup. A table presenting the results from the novel object test with the ball. Total duration: 90 min. “Type” indicates whether the individual was tested in the novel object test scenario with the ball as the first test (NO1) or the second test (NO2). ∆t out is the latency time before the individual left the carrier and entered the arena.

**Additional file 11.** Results from the novel arena test. A table presenting the results from the novel arena test. Total duration 900 s/15 min. ∆t out is the latency time before the individual left the carrier and entered the arena. ∆t X describes the latency time before the individual reached the respective zone. ∑ X describes the time spent in the respective zone. V X is the number of visits to the zone. Background is labelled R for hand-reared, rehabilitated, and W for wild.

## Data Availability

The datasets supporting the conclusions of this article are included within the article and its additional files.
