# Supplementary material for: An exploratory investigation of glucocorticoids, personality and survival rates in wild and rehabilitated hedgehogs (Erinaceus europaeus) in Denmark
Source: BMC Ecol Evol. 2021 May 22;21:96. doi: 10.1186/s12862-021-01816-7 (PMC8141197; doi:10.1186/s12862-021-01816-7)
Supplement: Supplementary file 1 — Additional file 1. Overview of individuals from cohort 1. The individuals of cohort 1 were all in care at a local hedgehog rehabilitation centre. [file 12862_2021_1816_MOESM1_ESM.pdf]

| Individual | Weight (g) | Sex | Background | Disease |
|---|---|---|---|---|
| 1 | 879 | Male | Rehabilitated | Lungworm (coughing) and myiasis |
| 2 | 953 | Male | Rehabilitated | Unknown |
| 3 | 908 | Male | Rehabilitated | Unknown |
| 4 | 1070 | Male | Rehabilitated | Unknown |
| 5 | 1082 | Female | Rehabilitated | Unknown |
| 6 | 911 | Male | Rehabilitated | Unknown |
| 7 | 751 | Female | Rehabilitated | Unknown |
| 8 | 915 | Female | Rehabilitated | Attacked by birds, stitched wound on eyelid, length of admission: 2 months |
| 9 | 868 | Male | Rehabilitated | Unknown |
| 10 | 704 | Female | Rehabilitated | Stitched wound in the corner of the mouth, length of admission: 2-3 months |
