# Supplementary figures and images for: An exploratory investigation of glucocorticoids, personality and survival rates in wild and rehabilitated hedgehogs (Erinaceus europaeus) in Denmark
Source: BMC Ecol Evol. 2021 May 22;21:96. doi: 10.1186/s12862-021-01816-7 (PMC8141197; doi:10.1186/s12862-021-01816-7)

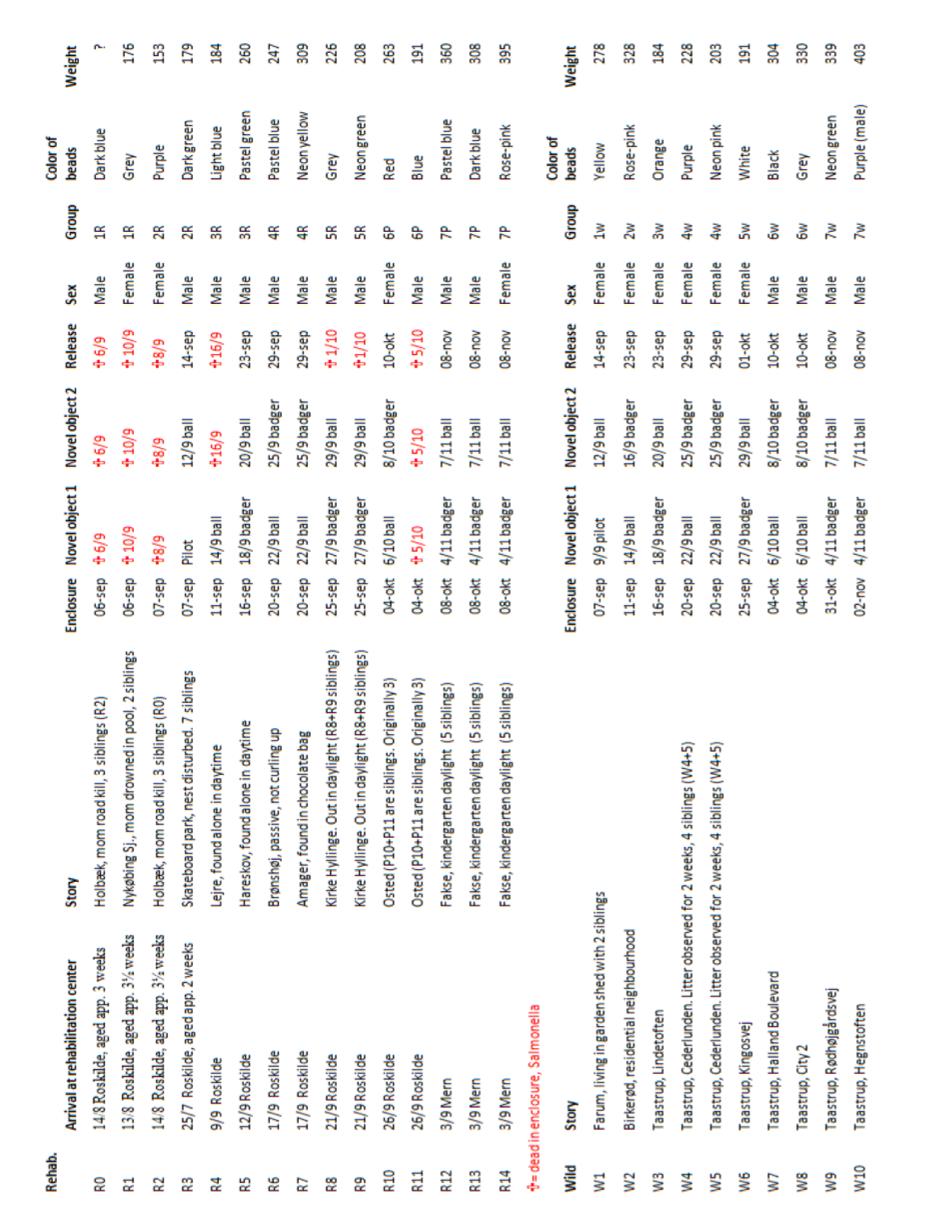

Supplement: Supplementary file 2 — Additional file 2. Overview of individuals in cohort 2. The column weight indicates the weight in grams of an individual when entering the study. [file 12862_2021_1816_MOESM2_ESM.png]

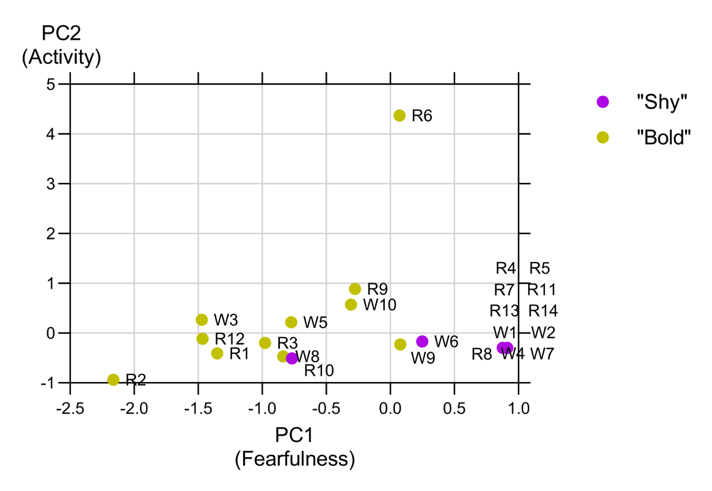

Supplement: Supplementary file 3 — Additional file 3. Novel arena tests: Distribution of individuals in PCA space. Note that the labelling of the axes is speculative. [file 12862_2021_1816_MOESM3_ESM.png]

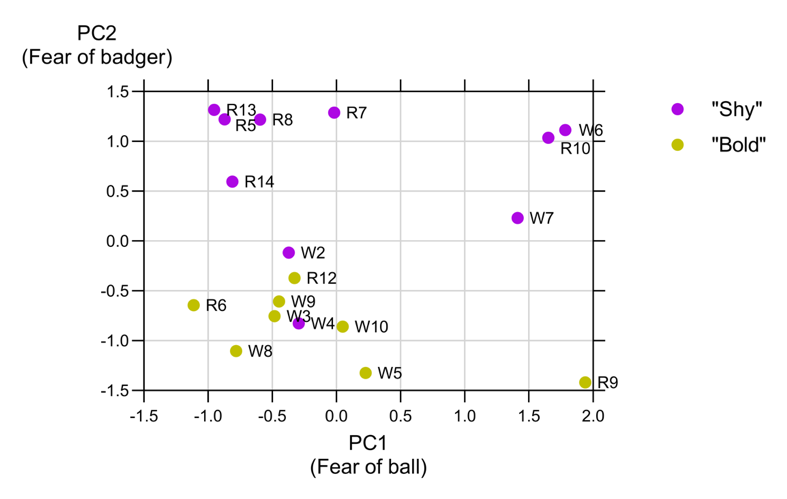

Supplement: Supplementary file 4 — Additional file 4. Novel object tests: Distribution of individuals in PCA space. Note that the labelling of the axes is speculative. [file 12862_2021_1816_MOESM4_ESM.png]

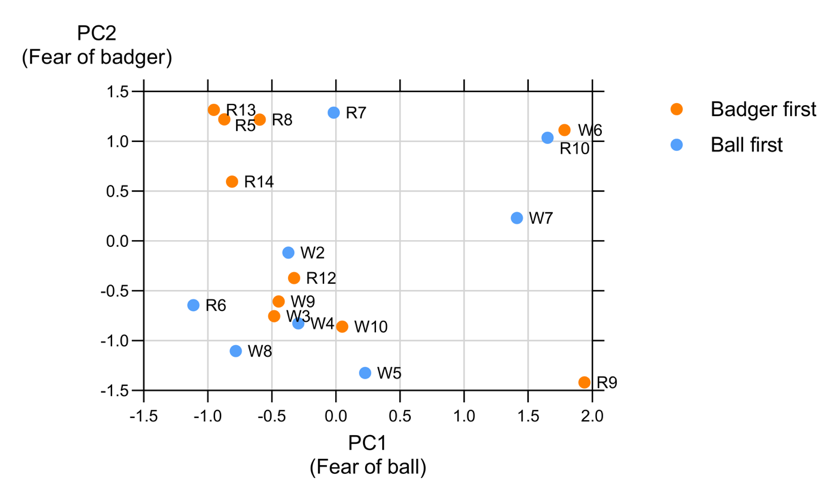

Supplement: Supplementary file 6 — Additional file 6. Novel object tests: Distribution of subjects in PCA space, labelled by order of test. No obvious effect of testing order can be seen. [file 12862_2021_1816_MOESM6_ESM.png]

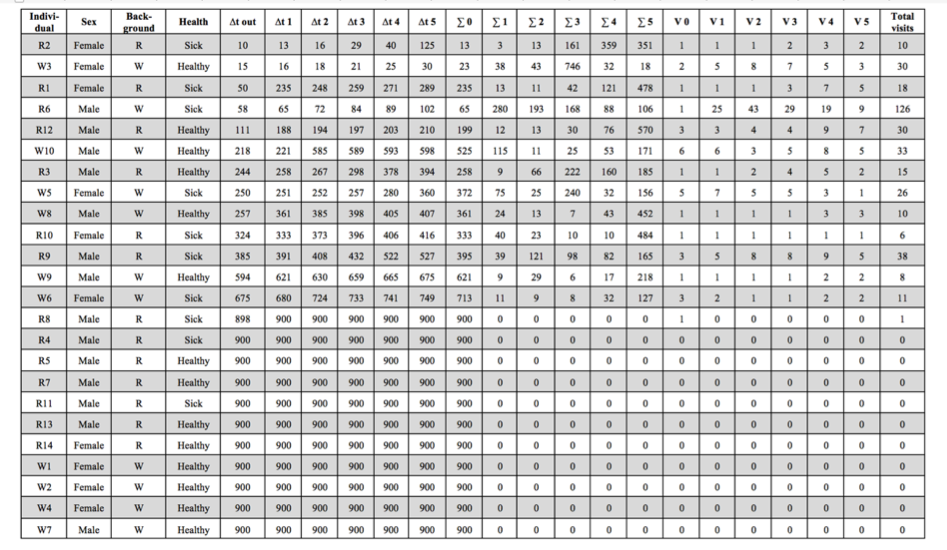

Supplement: Supplementary file 11 — Additional file 11. Results from the novel arena test. A table presenting the results from the novel arena test. Total duration 900 s/15 min. ∆t out is the latency time before the individual left the carrier and entered the arena. ∆t X describes the latency time before the individual reached the respective zone. ∑ X describes the time spent in the respective zone. V X is the number of visits to the zone. Background is labelled R for hand-reared, rehabilitated, and W for wild. [file 12862_2021_1816_MOESM11_ESM.png]
