# Supplementary material for: An exploratory investigation of glucocorticoids, personality and survival rates in wild and rehabilitated hedgehogs (Erinaceus europaeus) in Denmark
Source: BMC Ecol Evol. 2021 May 22;21:96. doi: 10.1186/s12862-021-01816-7 (PMC8141197; doi:10.1186/s12862-021-01816-7)
Supplement: Supplementary file 5 — Additional file 5. PC scores for the novel arena test data. [file 12862_2021_1816_MOESM5_ESM.pdf]

ID	PC1	PC2
R1	-1.351	-0.406
R2	-2.162	-0.939
R3	-0.978	-0.198
R4	0.912	-0.300
R5	0.912	-0.300
R6	0.072	4.374
R7	0.912	-0.300
R8	0.877	-0.298
R9	-0.276	0.886
R10	-0.769	-0.507
R11	0.912	-0.300
R12	-1.467	-0.114
R13	0.912	-0.300
R14	0.912	-0.300
W1	0.912	-0.300
W2	0.912	-0.300
W3	-1.472	0.265
W4	0.912	-0.300
W5	-0.775	0.217
W6	0.249	-0.167
W7	0.912	-0.300
W8	-0.837	-0.465
W9	0.077	-0.226
W10	-0.307	0.574
