# Supplementary material for: An exploratory investigation of glucocorticoids, personality and survival rates in wild and rehabilitated hedgehogs (Erinaceus europaeus) in Denmark
Source: BMC Ecol Evol. 2021 May 22;21:96. doi: 10.1186/s12862-021-01816-7 (PMC8141197; doi:10.1186/s12862-021-01816-7)
Supplement: Supplementary file 7 — Additional file 7. PC scores for the novel object tests’ data. [file 12862_2021_1816_MOESM7_ESM.pdf]

ID	Ball first	PC1	PC2
R5	0	-0.871	1.222
R8	0	-0.595	1.219
R9	0	1.939	-1.418
R12	0	-0.327	-0.370
R13	0	-0.953	1.316
R14	0	-0.811	0.597
W3	0	-0.482	-0.754
W6	0	1.784	1.114
W9	0	-0.447	-0.606
W10	0	0.048	-0.859
R6	1	-1.112	-0.645
R7	1	-0.018	1.289
R10	1	1.651	1.035
W2	1	-0.371	-0.116
W4	1	-0.293	-0.826
W5	1	0.228	-1.323
W7	1	1.411	0.231
W8	1	-0.781	-1.105
