# Supplementary material for: An exploratory investigation of glucocorticoids, personality and survival rates in wild and rehabilitated hedgehogs (Erinaceus europaeus) in Denmark
Source: BMC Ecol Evol. 2021 May 22;21:96. doi: 10.1186/s12862-021-01816-7 (PMC8141197; doi:10.1186/s12862-021-01816-7)
Supplement: Supplementary file 8 — Additional file 8. Personality. A table presenting the division of individuals into shy or bold based on their behaviour in the three personality tests. Shy behaviour is indicated by S and bold by B. [file 12862_2021_1816_MOESM8_ESM.pdf]

Individuals	Novel arena test	Novel object test with ball	Novel object test with badger	Shy (S) or bold (B)	Alive (A) or dead (D) post release
W1	S			**S**	D
W2	S	B	S	**S**	D
W3	B	B	B	**B**	D
W4	S	S	B	**S**	A
W5	B	S	B	**B**	D
W6	S	S	S	**S**	D
W7	S	S	S	**S**	A
W8	B	B	B	**B**	A
W9	B	B	B	**B**	A
W10	B	S	B	**B**	A
R3	B			**B**	D
R5	S	B	S	**S**	A
R6	B	B	B	**B**	D
R10	B	S	S	**S**	D
R12	B	S	B	**B**	A
R13	S	B	S	**S**	A
R14	S	B	S	**S**	A
R1	B			**B**	
R2	B			**B**	
R4	S			**S**	
R7	S	S	S	**S**	
R8	S	B	S	**S**	
R9	B	S	B	**B**	
R11	S			**S**	
