# Supplementary material for: An exploratory investigation of glucocorticoids, personality and survival rates in wild and rehabilitated hedgehogs (Erinaceus europaeus) in Denmark
Source: BMC Ecol Evol. 2021 May 22;21:96. doi: 10.1186/s12862-021-01816-7 (PMC8141197; doi:10.1186/s12862-021-01816-7)
Supplement: Supplementary file 9 — Additional file 9. Results from the novel object test with a badger setup. A table presenting the results from the novel object test with the badger. Total duration: 90 min. “Type” indicates whether the individual was tested in the novel object test scenario with the badger as the first test (NO1) or the second test (NO2). ∆t out is the latency time before the individual left the carrier and entered the arena. [file 12862_2021_1816_MOESM9_ESM.pdf]

Individual	Δt out (minutes out of 90 minutes)	Δt approach (max 50 cm. from object)	Nearest distance from object (cm)	Type	Sex	Background	Health
R6	0	45	25	NO2	Male	Rehabilitated	Sick
R9	7	13	0	NO1	Male	Rehabilitated	Sick
W5	8	8	0	NO2	Female	Wild	Healthy
W4	10	45	0	NO2	Female	Wild	Healthy
W8	10	19	0	NO2	Male	Wild	Healthy
W9	13	59	0	NO1	Male	Wild	Healthy
R12	14	78	0	NO1	Male	Rehabilitated	Healthy
R14	15	90	90	NO1	Female	Rehabilitated	Healthy
W2	16	80	25	NO2	Female	Wild	Healthy
W10	24	32	0	NO1	Male	Wild	Healthy
W3	28	35	0	NO1	Female	Wild	Healthy
W7	79	85	0	NO2	Male	Wild	Sick
R5	80	90	90	NO1	Male	Rehabilitated	Healthy
R8	80	90	90	NO1	Male	Rehabilitated	Sick
R10	80	90	90	NO2	Female	Rehabilitated	Sick
R13	89	90	90	NO1	Male	Rehabilitated	Healthy
R7	90	90	90	NO2	Male	Rehabilitated	Healthy
W6	90	90	90	NO1	Female	Wild	Sick
