# Supplementary material for: An exploratory investigation of glucocorticoids, personality and survival rates in wild and rehabilitated hedgehogs (Erinaceus europaeus) in Denmark
Source: BMC Ecol Evol. 2021 May 22;21:96. doi: 10.1186/s12862-021-01816-7 (PMC8141197; doi:10.1186/s12862-021-01816-7)
Supplement: Supplementary file 10 — Additional file 10. Results from the novel object test with a ball setup. A table presenting the results from the novel object test with the ball. Total duration: 90 min. “Type” indicates whether the individual was tested in the novel object test scenario with the ball as the first test (NO1) or the second test (NO2). ∆t out is the latency time before the individual left the carrier and entered the arena. [file 12862_2021_1816_MOESM10_ESM.pdf]

Individual	∆t out (minutes of out 90)	∆t approach (max 50 cm. From object)	Nearest distance from object (cm)	Type	Sex	Background	Health
R6	0	0	0	NO1	Male	Rehabilitated	Sick
R7	7	52	50	NO1	Male	Rehabilitated	Healthy
R13	9	9	0	NO2	Male	Rehabilitated	Healthy
W8	10	10	0	NO1	Male	Wild	Healthy
R14	12	27	0	NO2	Female	Rehabilitated	Healthy
W9	12	23	25	NO2	Male	Wild	Healthy
R5	13	13	0	NO2	Male	Rehabilitated	Healthy
R8	13	37	0	NO2	Male	Rehabilitated	Sick
W10	16	36	50	NO2	Male	Wild	Healthy
W3	20	26	0	NO2	Female	Wild	Healthy
W4	23	23	25	NO1	Female	Wild	Healthy
R12	25	43	0	NO2	Male	Rehabilitated	Healthy
W2	30	37	0	NO1	Female	Wild	Healthy
W5	34	51	25	NO1	Female	Wild	Sick
W7	49	90	90	NO1	Male	Wild	Healthy
R3	60	60	0	NO2	Male	Rehabilitated	Healthy
R10	80	90	90	NO1	Female	Rehabilitated	Sick
W1	80	89	0	NO2	Female	Wild	Healthy
R4	90	90	90	NO1	Male	Rehabilitated	Sick
R9	90	90	90	NO2	Male	Rehabilitated	Sick
W6	90	90	90	NO2	Female	Wild	Sick
